# Atlas of ticks (Acari: Argasidae, Ixodidae) in Germany: 2nd data update with focus on introduced and rarely observed species

**DOI:** 10.1007/s10493-026-01168-1

**Published:** 2026-07-23

**Authors:** Franz Rubel, Nina Król, Susanne Fischer, Cornelia Silaghi, Olaf Kahl

**Affiliations:** 1 Unit for Veterinary Public Health and Epidemiology, University of Veterinary Medicine Vienna, Vienna, Austria; 2 Institute of Animal Hygiene and Veterinary Public Health, University of Leipzig, Leipzig, Germany; 3 Institute of Infectiology, Friedrich-Loeffler Institute, Greifswald, Germany; 4 tick-radar GmbH, Berlin, Germany

**Keywords:** Tick map, Species distribution, Georeferenced tick locations

## Abstract

This atlas provides updated maps based on georeferenced tick locations of 22 species endemic in Germany, three regularly imported tick species and one species that was previously frequently imported into Germany. Thus, the current version of the atlas of ticks in Germany contains distribution maps for a total of 26 tick species with the following numbers of newly georeferenced tick locations: 1 *Argas reflexus*, 10 *Carios vespertilionis*, 3 *Dermacentor marginatus*, 53 *Dermacentor reticulatus*, 10 *Haemaphysalis concinna*, 7 *Haemaphysalis punctata*, 2 *Hyalomma aegyptium*, 111 *Hyalomma marginatum*, 39 *Hyalomma rufipes*, 2 *Ixodes arboricola*, 2 *Ixodes crenulatus* (syn. *Ixodes canisuga*), 27 *Ixodes frontalis*, 43 *Ixodes hexagonus*, 6 *Ixodes lividus*, 271 *Ixodes ricinus*, 1 *Ixodes rugicollis*, 5 *Ixodes trianguliceps*, 1 *Ixodes uriae*, 2 *Ixodes ventalloi*, 1 *Ornithodoros maritimus*, and 32 *Rhipicephalus sanguineus* sensu lato. *Ixodes inopinatus* has been removed from the previously included species list because more recent studies have shown that these were misidentifications. The rabbit tick *I. ventalloi*, the argasid seabird tick *O. maritimus* and the formerly frequently imported tortoise tick *Hy. aegyptium*, however, have been included in the current atlas of ticks in Germany. In total, the digital dataset available for Germany is updated by 629 new tick locations. These records increase the number of tick species mapped in the federal state Saxony by six, those in Hamburg by five, those in Brandenburg by four, those in Schleswig-Holstein by three, those in Baden-Wuerttemberg, Bavaria and Thuringia by two each, and those in Bremen, Northrhine-Westphalia, Rhineland Palatinate and Saarland by one each. Of the 26 tick species ever detected in Germany, 21 were observed in the period 2001–present. Thus, the second data update of the atlas of ticks in Germany provides the most comprehensive digital dataset to date on the distribution of tick species in Germany.

**Supplementary Information:**

The online version contains supplementary material available at 10.1007/s10493-026-01168-1.

## Introduction

The atlas of ticks in Germany by Rubel et al. (2021, 2023) included 24 tick species, two species of Argasidae and 22 species of Ixodidae. The occurrence and distribution of all tick species was visualized in maps based on georeferenced locations and made available in the form of digital data. Starting with the first digital dataset by Rubel et al. (2014), 4346 locations have been published in the atlas of ticks in Germany so far.

Recently, the occurrence of an argasid tick species, the seabird tick *Ornithodoros maritimus*, was documented for the first time in Germany (Rollins et al. 2025). Together with the previously known findings of the rabbit tick *Ixodes ventalloi* (Walter et al. 1979; Petney et al. 1996) and the formerly frequently imported tortoise tick *Hyalomma aegyptium* (Rubel 2025), three species have been newly added to the atlas of ticks in Germany. Conversely, one ixodid tick species, *Ixodes inopinatus* (Estrada-Peña et al. 2014), must be removed as recent research indicates that there were several misidentifications (Laatamna et al. 2025; Estrada-Peña et al. 2025b).

Meanwhile, the technical prerequisites for the implementation of citizen science studies have also been provided. The ticks reported by citizen science studies predominantly comprise the common species *Ixodes ricinus*, *Dermacentor reticulatus* and *Dermacentor marginatus* (Springer et al. 2022; Köppen et al. 2025; Fischer et al. 2025b). These studies provide only very few new insights into the distribution of *I. ricinus*, which occurs throughout Germany. However, citizen science studies confirm that *D. reticulatus* is now also found throughout Germany and that *D. marginatus* has a larger distribution area than previously known from Walter et al. (2016). Other recently published citizen science studies also include numerous new finding spots of *Hyalomma marginatum* and *Hyalomma rufipes*, which are imported by migratory birds from the south each spring (Chitimia-Dobler et al. 2024b) as well as *Rhipicephalus sanguineus* sensu lato occasionally imported by dogs coming from the Mediterranean or other southern countries (Fachet-Lehmann et al. 2025). Newly digitized locations of less frequently observed tick species such as *Haemaphysalis concinna*, *Ixodes arboricola*, *Ixodes frontalis*, *Ixodes lividus* and *Ixodes trianguliceps* originate from scientific field studies (Król et al. 2024; Fischer et al. 2025a; Krüger et al. 2025) or archives such as the Zoological Museum Hamburg (Weidner 1954). In addition, there are new unpublished tick findings from the authors and from colleagues, such as the observation of the pigeon tick *Argas reflexus* (Haas 2024). The aforementioned new tick findings justify a data update that will further improve the mapping of tick species in Germany. For this purpose, the newly georeferenced locations and the updated maps are presented here.

## Data and methods

The data used here are georeferenced tick locations in Germany described by Rubel et al. (2014, 2021, 2023) supplemented by 629 new records. The geographical coordinates of the new tick locations are provided in the supplement together with an indication of their accuracy and the sources. The coordinates are given in decimal degrees with an established measure of accuracy identical to those previously introduced for the first German map of georeferenced ixodid tick locations (Rubel et al. 2014) and also used in other studies (Estrada-Peña et al. 2025a).

**Fig. 1:**
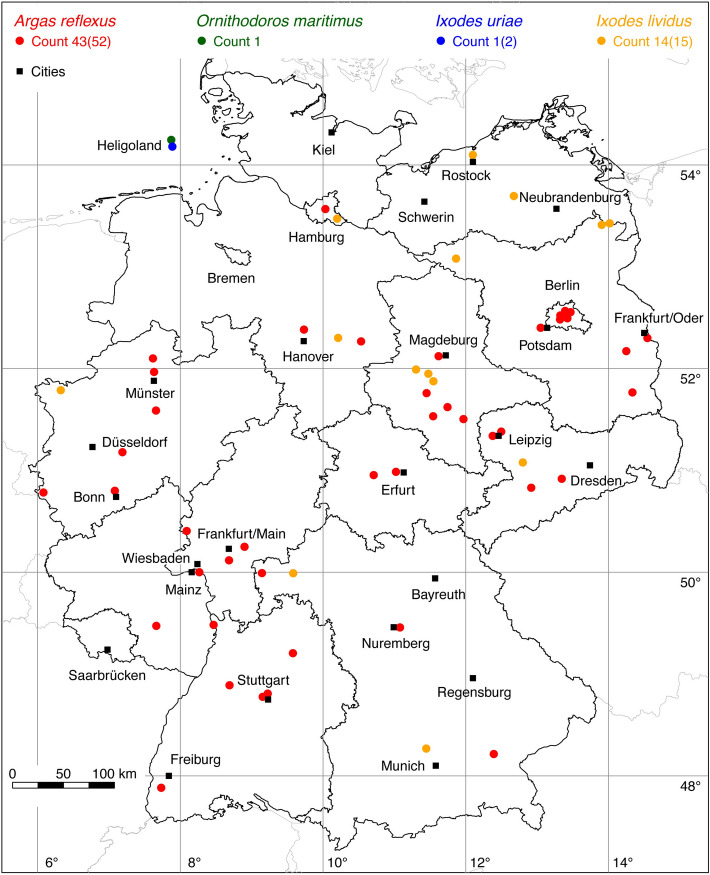
Recorded locations of *Argas reflexus*, *Ornithodoros maritimus*, *Ixodes uriae* and *Ixodes lividus* in Germany.

**Fig. 2:**
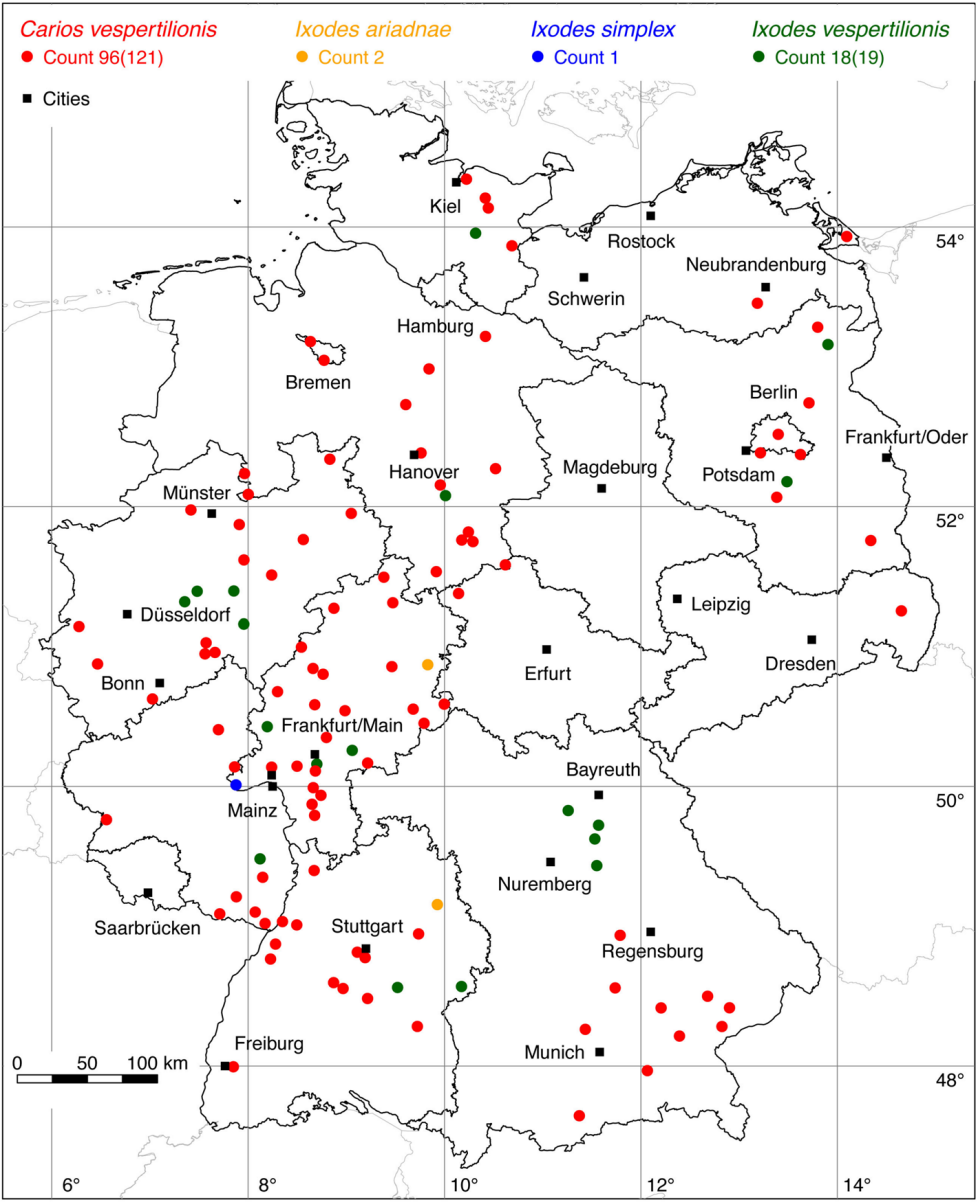
Recorded locations of *Carios vespertilionis*, *Ixodes ariadnae*, *Ixodes simplex* and *Ixodes vespertilionis* in Germany.

**Fig. 3:**
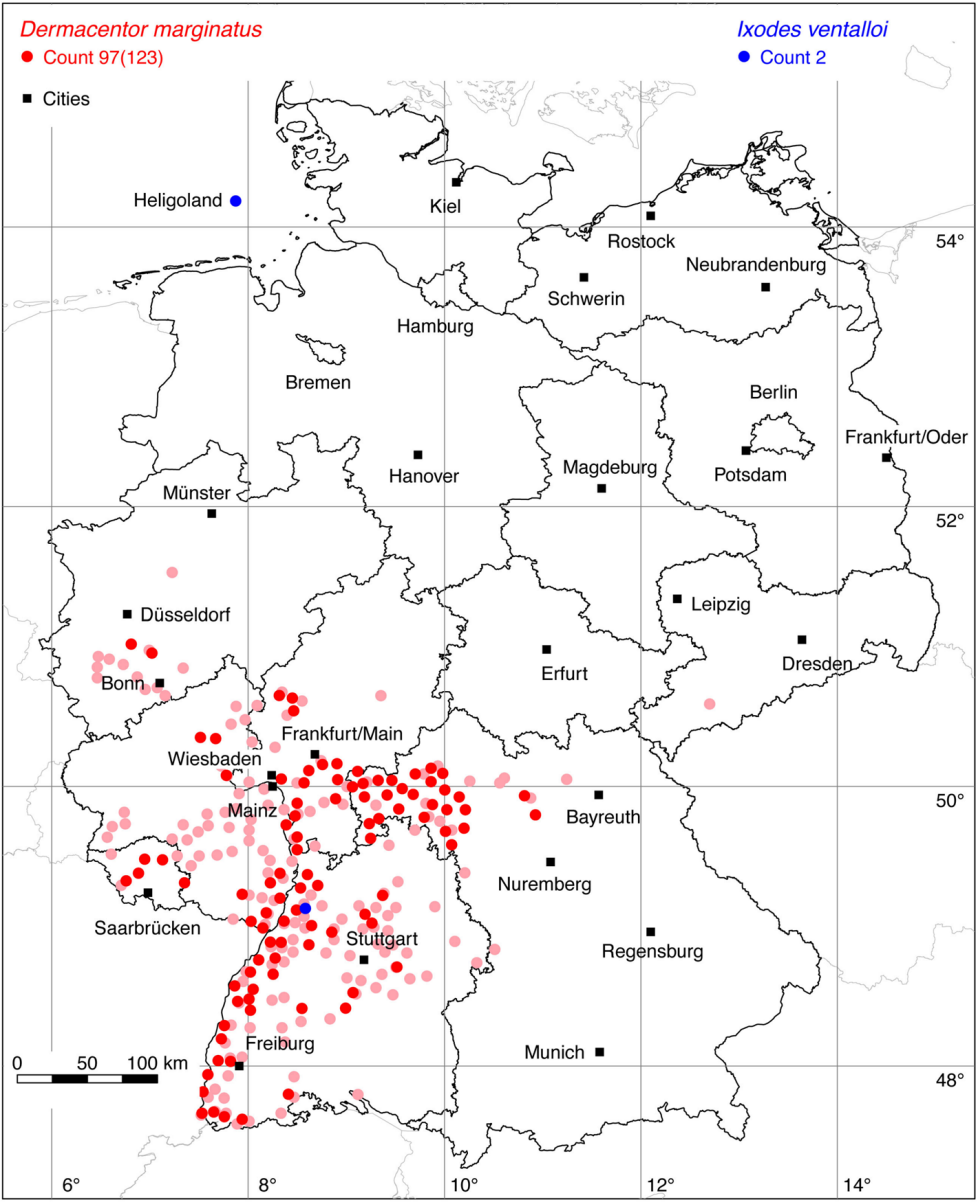
Recorded locations of *Dermacentor marginatus* and *Ixodes ventalloi* in Germany. *Dermacentor marginatus* findings (red) are complemented by a selection of citizen science reports (light red).

**Fig. 4:**
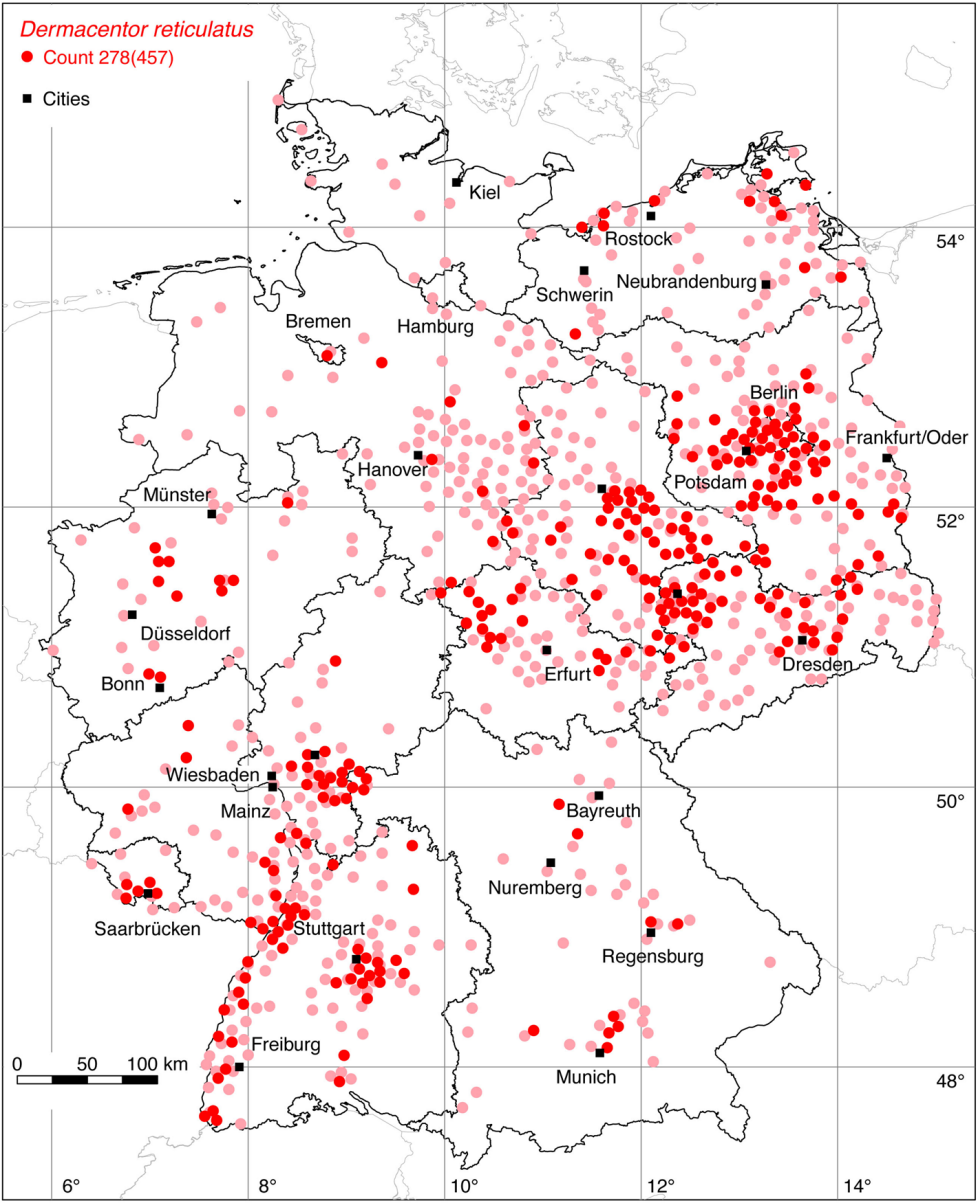
Recorded locations of *Dermacentor reticulatus* in Germany (red) are complemented by a selection of citizen science reports (light red).

**Fig. 5:**
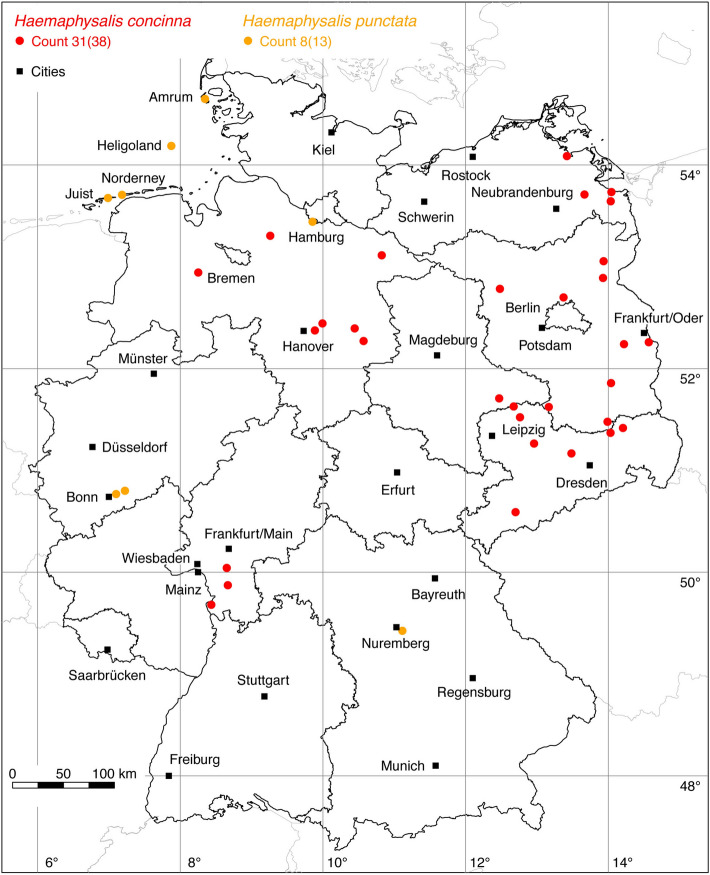
Recorded locations of *Haemaphysalis concinna* and *Haemaphysalis punctata* in Germany.

**Fig. 6:**
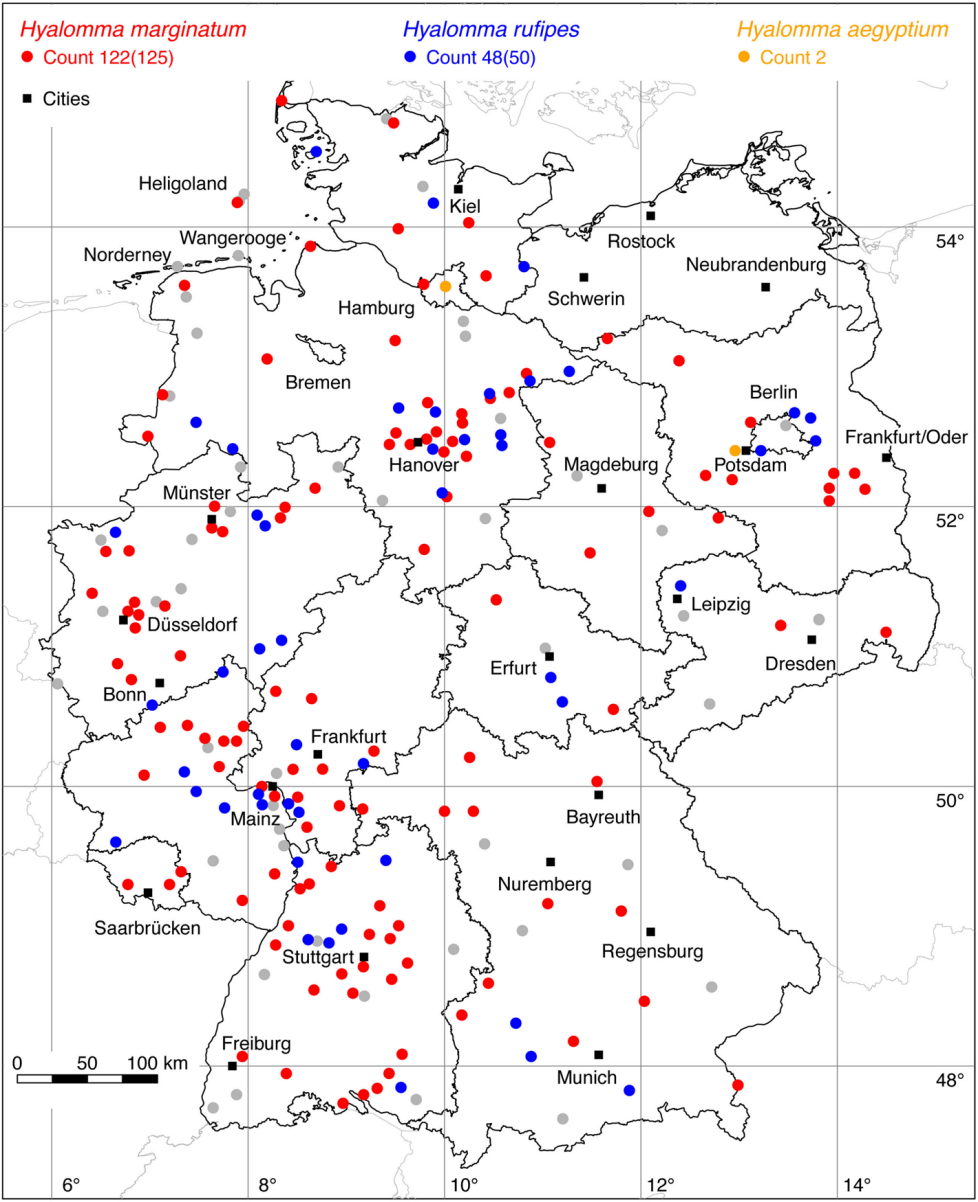
Recorded locations of *Hyalomma marginatum*, *Hyalomma rufipes* and *Hyalomma aegyptium* in Germany. *Hyalomma* spp. are depicted in grey. These species are not endemic in Germany, but are continuously introduced.

**Fig. 7:**
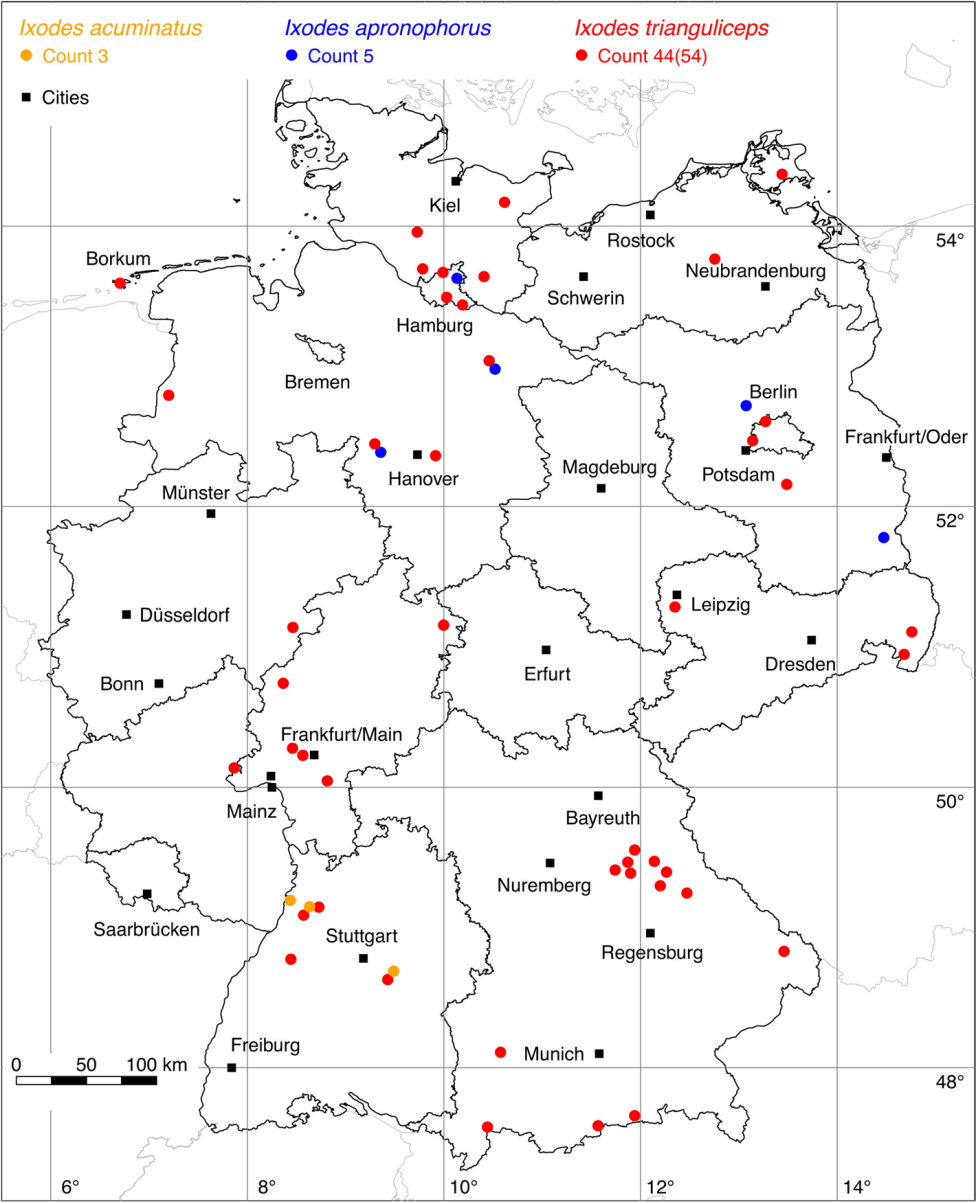
Recorded locations of *Ixodes acuminatus*, *Ixodes apronophorus* and *Ixodes trianguliceps* in Germany.

**Fig. 8:**
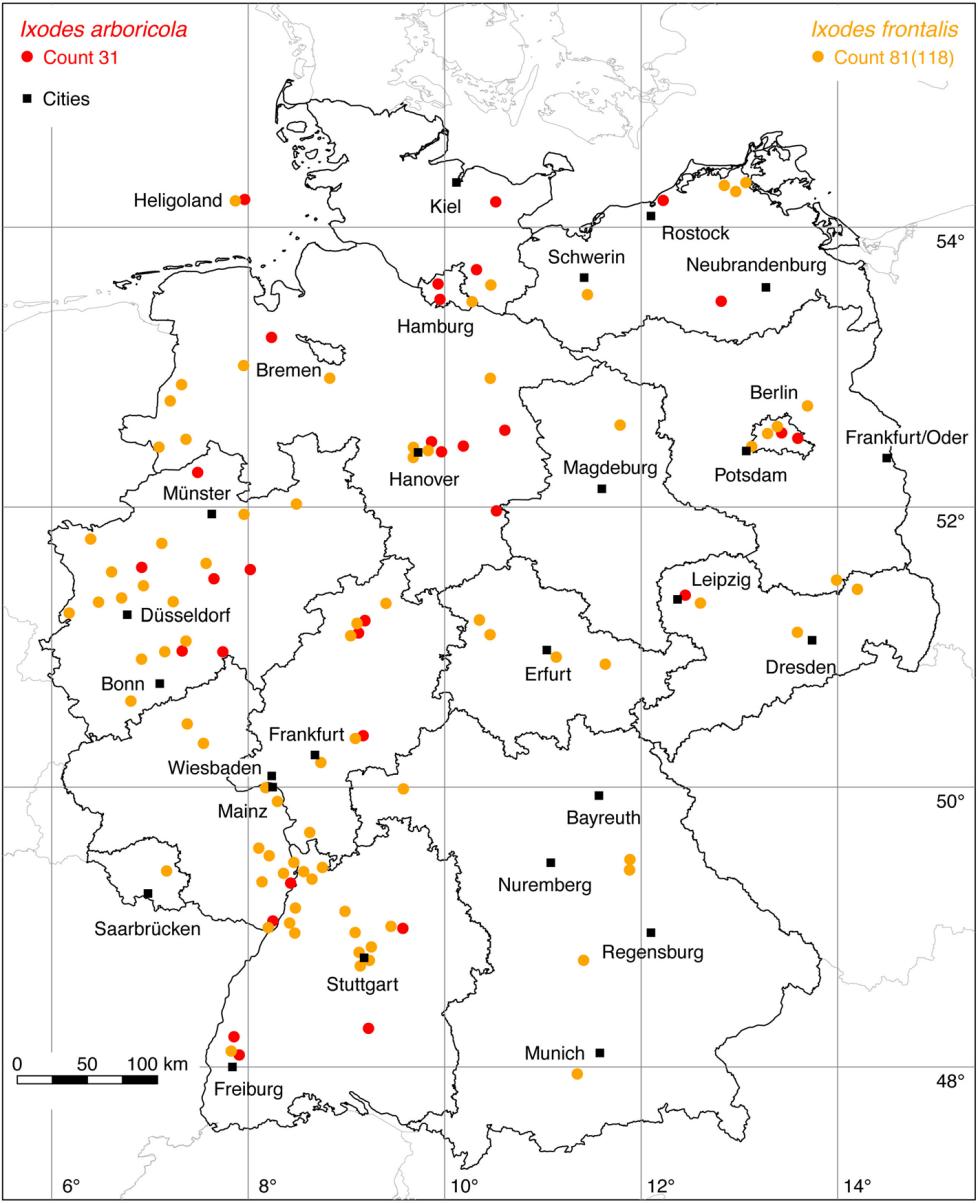
Recorded locations of *Ixodes arboricola* and *Ixodes frontalis* in Germany.

**Fig. 9:**
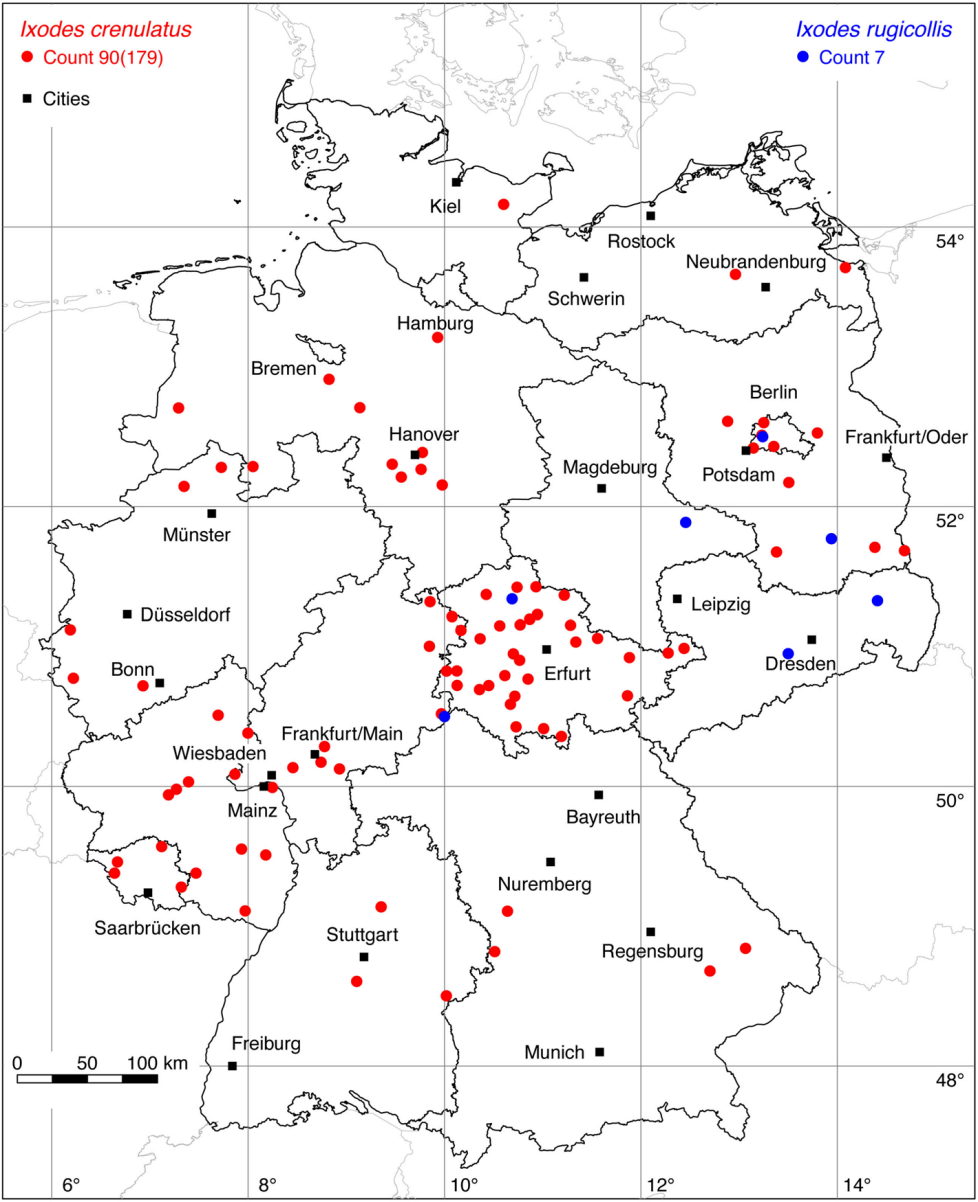
Recorded locations of *Ixodes crenulatus* and *Ixodes rugicollis* in Germany.

**Fig. 10:**
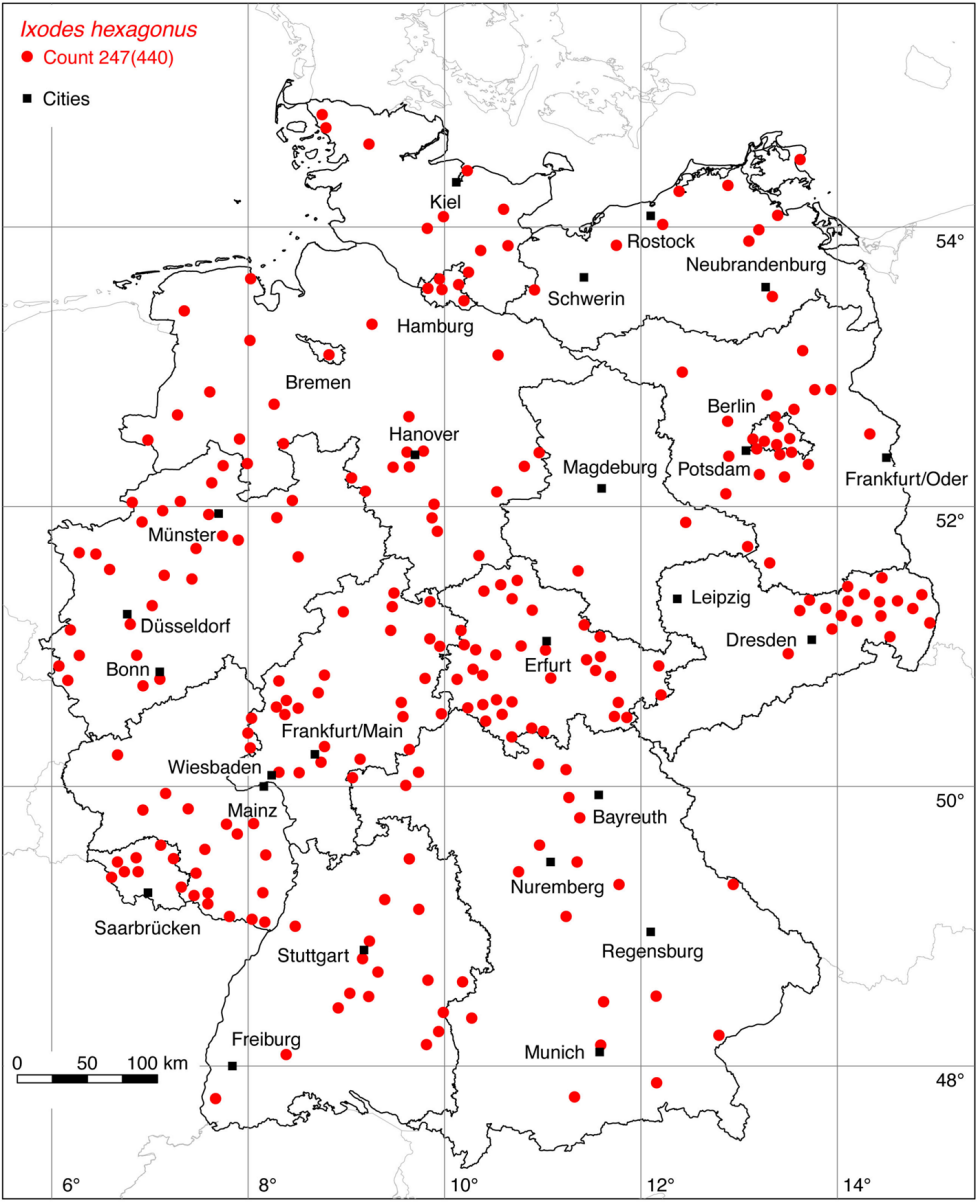
Recorded locations of *Ixodes hexagonus* in Germany.

**Fig. 11:**
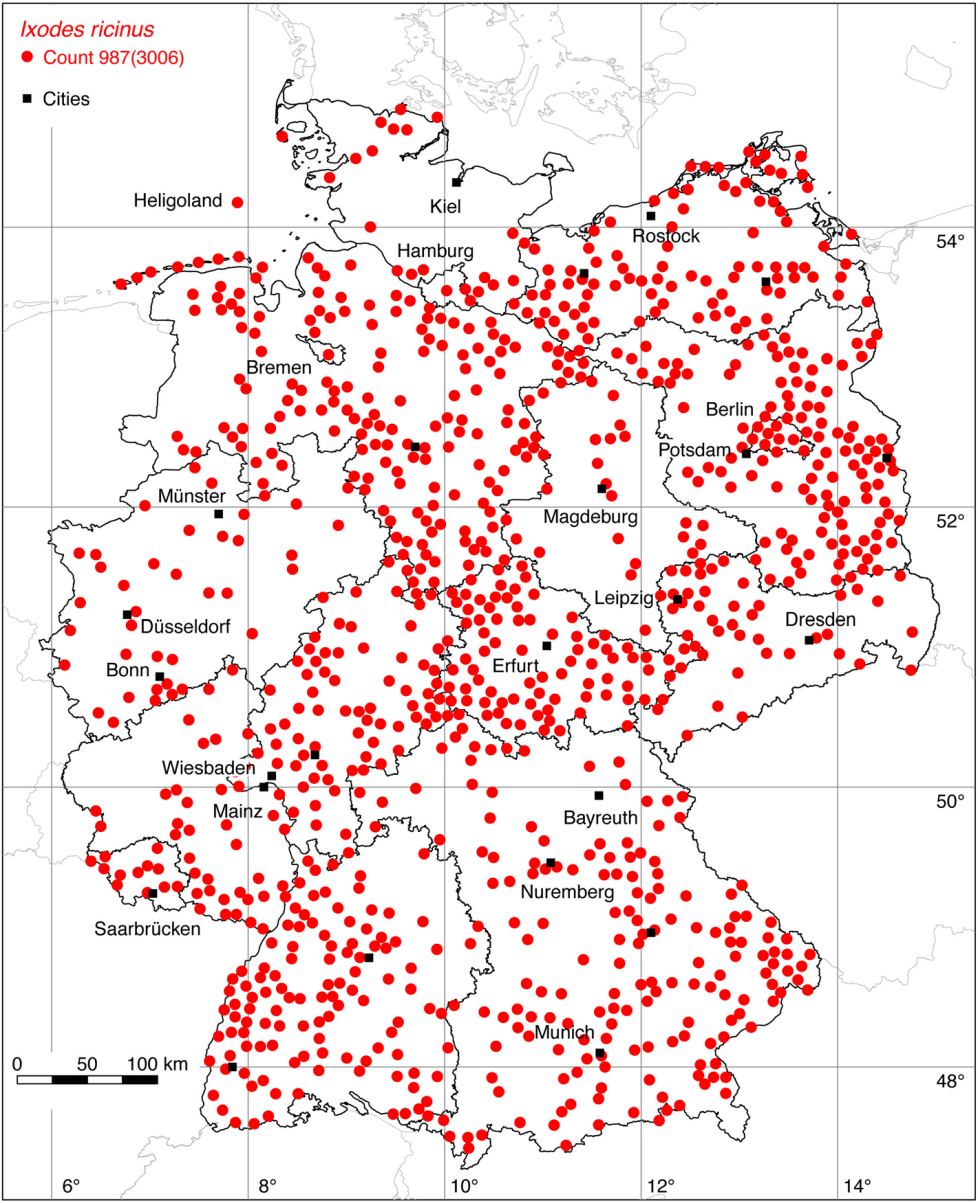
Recorded locations of *Ixodes ricinus* in Germany

**Fig. 12:**
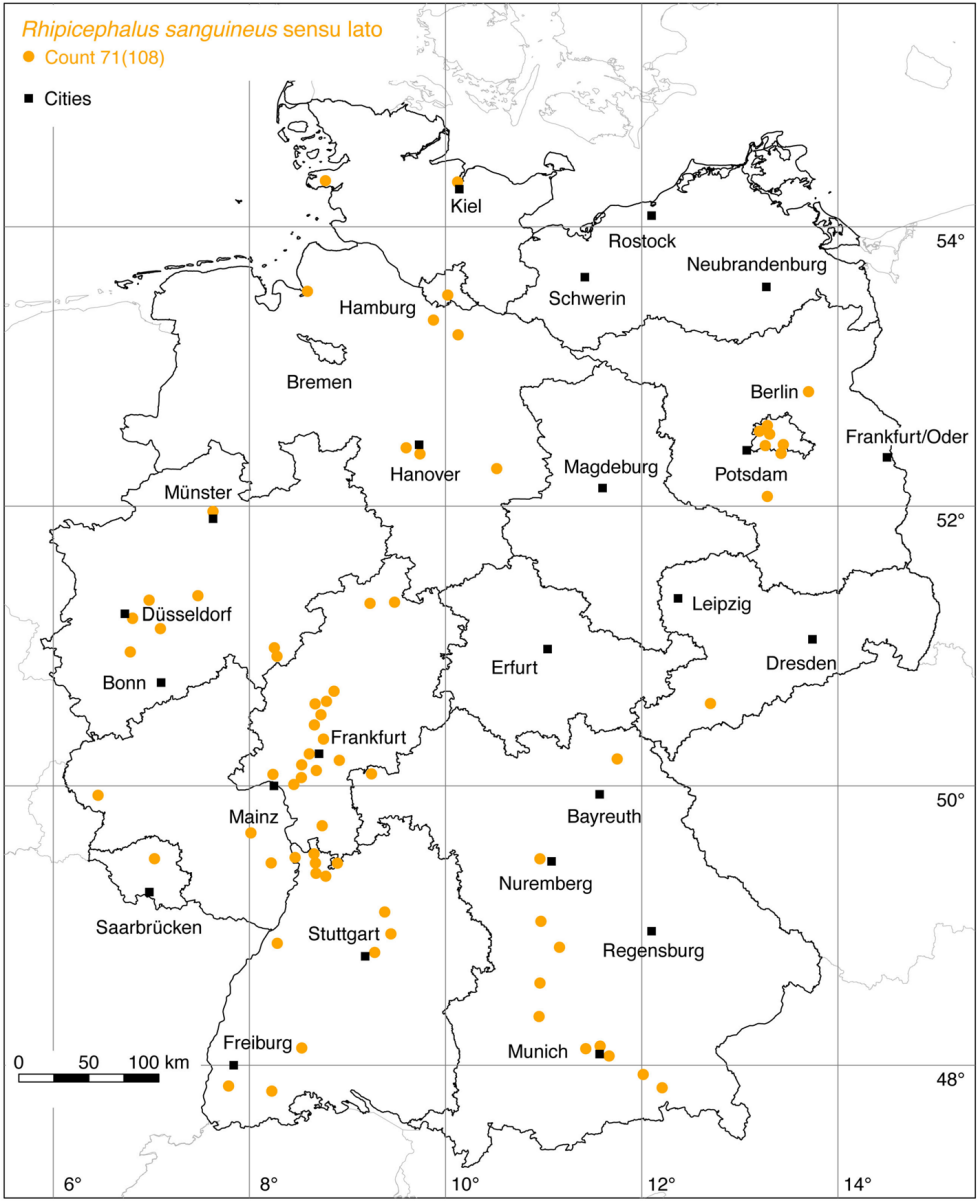
Recorded locations of *Rhipicephalus sanguineus* sensu lato in Germany. Species of that complex are not endemic in Germany, but are continuously introduced.

The tick locations are mapped using R, a language and environment for statistical computing (Development 2021). Artificial data clusters caused by single studies were reduced using a random selection and a thinning algorithm (Aiello-Lammens et al. 2019). For example, the newly georeferenced tick locations of the study by Chitimia-Dobler et al. (2024b) significantly increased the number of *Hy. marginatum* and *Hy. rufipes* reports. However, only 122 out of 125 known *Hy. marginatum* locations and 48 out of 50 known *Hy. rufipes* locations were mapped to avoid overlapping location points. This makes the maps clearer. The maps for the individual tick species (Figs. 1–12) therefore not only show the number of tick locations mapped, but also the total number of available tick locations in brackets.

Tick species for which only a limited number of locations are known are shown in collective maps. This applies, for example, to the *Hyalomma* species *Hy. aegyptium*, *Hy. marginatum* and *Hy. rufipes*, which are all non-endemic in Germany but have regularly been introduced. Some of the *Hyalomma* ticks found could not be identified to the species level. Therefore, the category *Hyalomma* spp. is shown for the first time in this map of the tick atlas of Germany.

Also for the first time, some hundreds of *Dermacentor* locations from citizen science studies were included in the corresponding maps, shown in lighter colours to highlight the impact of this new data source. In contrast to all other data, these locations were not included in the supplement, but reference was made to the original sources. The ticks collected by volunteers across Germany between 2021 and 2024 (Köppen et al. 2025) originate from the citizen science project *Ticks and their Pathogens in Climate Change* (*Zecken und ihre Pathogene im Klimawandel*, ZEPAK) and can be viewed on an interactive map (https://www.zepak-rki.de/). The citizen science study by the Friedrich Loeffler-Institute (FLI) reveals new locations of *D. reticulatus* in Northern Germany for the period 2023 to 2024 (Fischer et al. 2025b), supplemented by new locations from 2025.

## Results and discussion

The results of this study are updated maps of the geographic distribution of 23 tick species, based on the previous version of the atlas of ticks in Germany (Rubel et al. 2023). It should be noted that one tick species, *I. inopinatus* (Estrada-Peña et al. 2014), has been removed from the species list, as recent studies have shown that all those cases were misidentifications (Rollins et al. 2023; Laatamna et al. 2025; Estrada-Peña et al. 2025b). Consequently, *I. inopinatus* must also be removed from the *Annotated Checklist of the Ticks of Germany* (Petney et al. 2015) and all publications regarding its occurrence outside the Mediterranean region must be critically reviewed.

Another change concerns the fox tick *Ixodes canisuga* Johnston, 1849, which here, in contrast to the previous atlas of ticks in Germany (Rubel et al. 2021, 2023), is considered a junior synonym of *Ixodes crenulatus* Koch, 1844. We follow herein the view of authors from Eastern and Central Europe (Cerny 1969; Siuda 1993; Siuda and Sebesta 1997; Kolonin 2009; Karbowiak et al. 2020; Hornok et al. 2021), who postulated that *I. canisuga* and *I. crenulatus* are synonymous, i.e. a single species. This is confirmed by the current phylogenetic analysis of Wang et al. (2026), which revealed a clear genetic relationship between *I. canisuga* and *I. crenulatus*, clustering them into a single major branch.

Findings of the rabbit tick *I. ventalloi* (Petney et al. 1996), the seabird tick *O. maritimus* (Rollins et al. 2025) and the tortoise tick *Hy. aegyptium* (Rubel 2025), however, have been newly included in the current atlas of ticks in Germany. The improvements resulting from the data update are summarized in Table 1. That table shows the occurrence of now 26 tick species in the 16 federal states of Germany. The most frequently observed tick, *I. ricinus* (Kahl and Gray 2023), is widespread throughout Germany. This update also made it possible for the first time to map the second most frequently observed tick species *D. reticulatus* as well as the hedgehog tick *I. hexagonus* in every federal state. This is followed by *I. frontalis*, which has been detected in 14 federal states, although it can be assumed that this bird tick is present in all states. However, the findings designated as Bremen and Hamburg (Król et al. 2026) are located somewhat outside the political boundaries of these federal states. Generally, *I. frontalis* might be much more common than the existing finding spots indicate. It is not a typical exophilic tick such as *I. ricinus*, and it seems as if the larval and the nymphal stages are mainly active in winter (Kahl et al. 2019; Plantard et al. 2021, 2026) when only few researchers are systematically flagging for questing/unfed ticks in green areas. At the end of Table 1 are five tick species listed that occur only in one federal state. These include the two seabird ticks, the ixodid tick *Ixodes uriae* and the argasid tick *O. maritimus*, on the North Sea island of Heligoland. Accordingly, the second data update increases the number of tick species mapped in the federal state Saxony by six, those in Hamburg by five, those in Brandenburg by four, those in Schleswig-Holstein by three, those in Baden-Wuerttemberg, Bavaria and Thuringia by two each, and those in Bremen, Northrhine-Westphalia, Rhineland Palatinate and Saarland by one each.

**Table 1:**
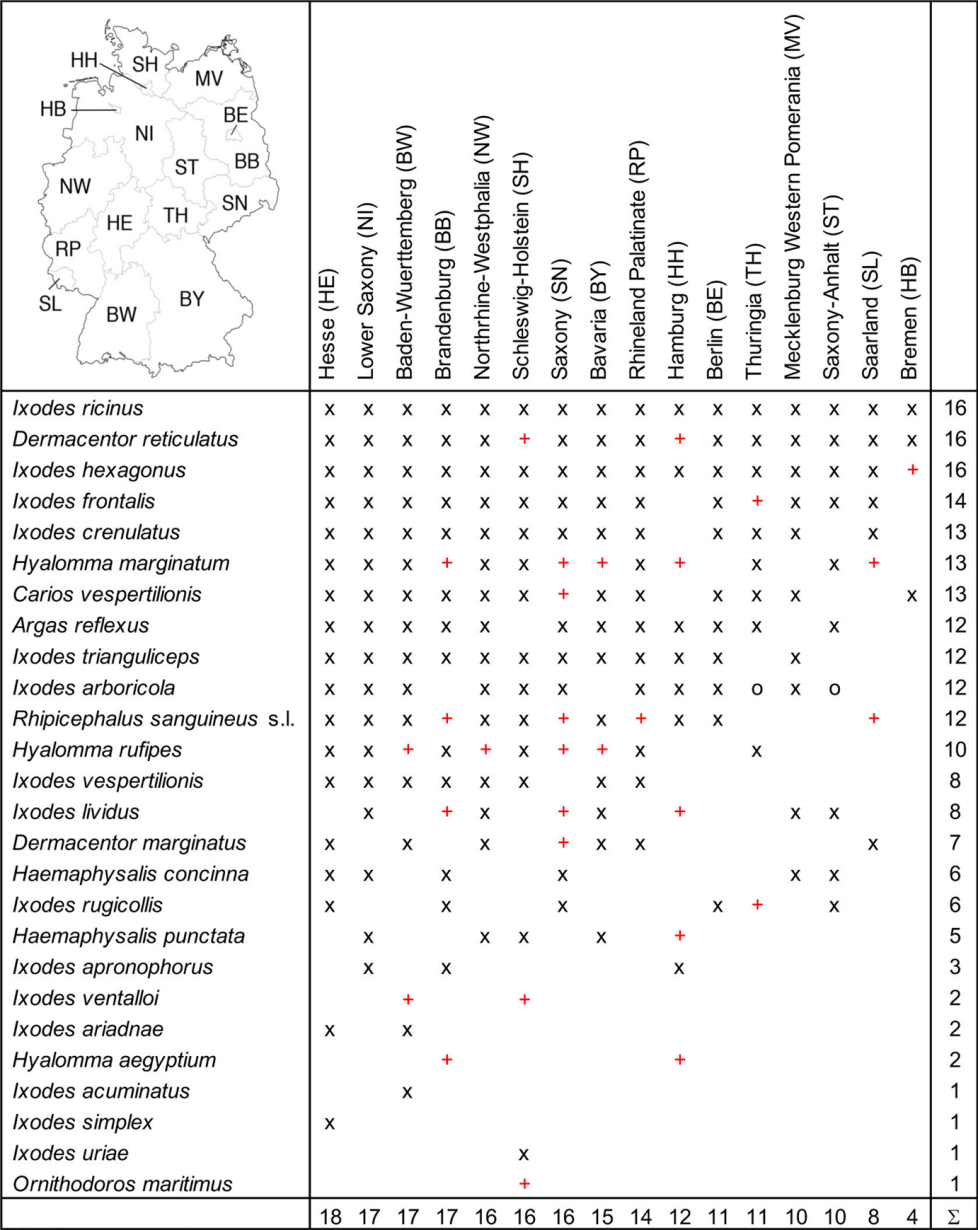
Occurrence of 26 tick species (Acari: Argasidae, Ixodidae) in the 16 German federal states: x) georeferenced locations already mentioned in Rubel et al. (2021, 2023), +) locations of the 2nd data update, and o) documented at the level of federal states (new data in red)

All the herein recorded tick species are presented below with a brief summary of the numbers of updated locations compiled for this study. For information on the biology, hosts, as well as medical and veterinary importance of the tick species the reader is referred to Petney et al. (2012, 2015). Only for the three newly included tick species the global distribution will be briefly described, as was done for all other tick species in the first version of the atlas of ticks in Germany (Rubel et al. 2021).

### *Argas (Argas) reflexus* (Fabricius)

The following new location was added to the distribution map of the pigeon tick *A. reflexus*: 1 (Haas 2024). For another recent *A. reflexus* find in Berlin (Köppen et al. 2025), no coordinates are available, which is why it could not be considered here. However, this finding confirms the reports about the pigeon tick in Berlin from the 1980 s and 1990 s (Dautel et al. 1999; Rubel et al. 2022), i.e. it still occurs there, but probably in much lower numbers today. A total of 43 out of 52 known *A. reflexus* locations is depicted in Fig. 1.

### *Carios (Carios) vespertilionis* (Latreille)

The following ten locations were added to the distribution map of the short-legged bat tick *C. vespertilionis*: 10 (Fritzsche et al. 2023). A total of 96 out of 121 known locations of the soft tick *C. vespertilionis* is mapped in Fig. 2.

### *Dermacentor marginatus* (Sulzer)

The following three locations were added to the distribution map of the ornate sheep tick *D. marginatus*: 3 (Krüger et al. 2025). The findings documented in scientific papers were supplemented with a selection of citizen science data from Springer et al. (2022) and Köppen et al. (2025). A total of 97 out of 123 recorded locations, supplemented by citizen science records (light red), is mapped in Fig. 3.

### *Dermacentor reticulatus* (Fabricius)

The following 53 locations were added to the distribution map of the ornate dog tick *D. reticulatus*: 3 OK, 10 CS, 3 (Bauch and Danner 1988), 1 (Heddergott 2009), 1 (Bröker et al. 2020), 1 (Ott et al. 2020), 2 (Arz et al. 2023), 1 (Ebert et al. 2023), 1 (Weber 2023), 12 (Król et al. 2024), 1 (Fischer et al. 2025a), 1 (Krüger et al. 2025), 16 (Lassen et al. 2025). These findings documented in scientific papers and others still unpublished were supplemented with a selection of citizen science data from Springer et al. (2022), Köppen et al. (2025) and Fischer et al. (2025b). A total of 278 out of 457 recorded locations, supplemented by citizen science records (light red), is depicted in Fig. 4.

### *Haemaphysalis (Haemaphysalis) concinna* Koch

The following ten locations were added to the distribution map of *Ha. concinna*: 1 CS, 5 (Król et al. 2024),1 (Fischer et al. 2025a), 3 (Köppen et al. 2025). A total of 31 out of 38 known locations is mapped in Fig. 4.

### *Haemaphysalis (Aboimisalis) punctata* Canestrini and Fanzago

The following seven locations were added to the distribution map of the red sheep tick *Ha. punctata*: 1 (Weidner 1954), 2 (Melfens 1978), 4 (Rollins et al. 2025). A total of 8 out of 13 known locations is depicted in Fig. 5.

### *Hyalomma aegyptium* (Linnaeus)

The tortoise tick *Hy. aegyptium* is included for the first time in the atlas of ticks in Germany and therefore its distribution is briefly described analogously to all other species in the earlier versions of the atlas. The global distribution of *Hy. aegyptium*, for which the synonym *Hyalomma syriacum* Schulze 1920 was used for some time, extends from Morocco in Northwestern Africa to Kyrgyzstan in Central Asia between 10$^{\circ }$ W–73$^{\circ }$ E and 28–46$^{\circ }$ N (Rubel 2025). Germany is therefore clearly outside the natural distribution area of *Hy. aegyptium* and all documented findings refer to ticks collected from imported tortoises of the genus *Testudo*, which are the predominant hosts of all parasitic life stages (Hoogstraal 1956). Mainly Greek tortoises, also commonly known as the spur-thighed tortoises *Testudo graeca* from the Mediterranean region were exported in large numbers to Central and Northern Europe from the 1950 s onwards, traded and kept as pets (Rubel 2025). With the entry into force of the *Washington Convention on International Trade in Endangered Species of Wild Fauna and Flora* (CITES) in West Germany in 1976, the market for tortoises of the genus *Testudo* gradually collapsed and *Hy. aegyptium* apparently disappeared from Germany. However, no studies of ticks on illegally imported reptiles are available. Historical findings of *Hy. aegyptium* are documented in two terrariums and a garden in Hamburg (Weidner 1954) as well as in a pet shop in Potsdam in the former German Democratic Republic (Negrobov and Borodin 1964). These two locations are depicted in Fig. 6.

### *Hyalomma (Euhyalomma) marginatum* Koch

The following 111 locations were added to the map of *Hy. marginatum*: 111 (Chitimia-Dobler et al. 2024b). A total of 122 out of 125 known locations is depicted in Fig. 6.

### *Hyalomma (Euhyalomma) rufipes* Koch

The following 39 locations were added to the distribution map of the hairy or coarse bont-legged *Hyalomma* tick, *Hy. rufipes*: 39 (Chitimia-Dobler et al. 2024b). A total of 48 out of 50 known locations is depicted in Fig. 6.

### *Ixodes (Ixodes) acuminatus* Neumann

No locations were added to the distribution map of *I. acuminatus*. A total of three known locations is depicted in Fig. 7.

### *Ixodes (Ixodes) apronophorus* Schulze

No locations were added to the distribution map of *I. apronophorus*: A total of five locations is depicted in Fig. 7.

### *Ixodes (Pholeoixodes) arboricola* Schulze and Schlottke

The following two old locations were added to the distribution map of *I. arboricola*: 2 (Weidner 1954). A total of 31 known locations is depicted in Fig. 8.

### *Ixodes (Pholeoixodes) ariadnae* Hornok

No locations were added to the distribution map of *I. ariadnae*. A total of two known locations is depicted in Fig. 2.

### *Ixodes (Pholeoixodes) crenulatus* Koch

The following two locations were added to the distribution map of the endophilic fox tick *I. crenulatus*: 2 (Schantz et al. 2025). A total of 90 out of 179 known locations is depicted in Fig. 9.

### *Ixodes (Trichotoixodes) frontalis* (Panzer)

The following 27 locations were added to the distribution map of *I. frontalis*: 8 OK, 1 (Weidner 1954), 1 (Rollins et al. 2021), 2 (Król et al. 2024), 1 (Krüger et al. 2025), 2 (Lassen et al. 2025), 12 (Król et al. 2026). A total of 81 out of 119 known locations is depicted in Fig. 8.

### *Ixodes (Pholeoixodes) hexagonus* Leach

The following 43 locations were added to the distribution map of the endophilic hedgehog tick *I. hexagonus*: 11 (Weidner 1954), 5 (Liebisch et al. 1989), 1 (Heddergott 2009), 1 (Probst et al. 2023), 2 (Köppen et al. 2025), 2 (Schantz et al. 2025), 21 (Fischer et al. 2025b). A total of 247 out of 440 known locations is depicted in Fig. 10.

### *Ixodes (Pholeoixodes) lividus* Koch

The following six locations were added to the distribution map of the sand martin tick *I. lividus*: 2 (Weidner 1954), 1 (Wadewitz 1955), 3 (Schmidt 1990). A total of 14 out of 15 known locations are depicted in Fig. 1.

### *Ixodes (Ixodes) ricinus* (Linnaeus)

The following 271 locations were added to the distribution map of the castor bean tick *I. ricinus*: 19 OK, 1 (Herold 1939), 82 (Danailov et al. 1965), 1 (Melfens 1978), 2 (Ullmann et al. 1984), 5 (Bark 1986), 7 (Walter 2000), 1 (Heddergott 2009), 8 (Scheid et al. 2016), 3 (Ott et al. 2020), 1 (Rollins et al. 2021), 56 (Borde et al. 2022), 13 (Arz et al. 2023), 15 (Król et al. 2024), 2 (Krüger et al. 2025), 50 (Lassen et al. 2025), 4 (Rollins et al. 2025), 1 (Fischer et al. 2025a). A total of 987 out of 3,006 known locations is depicted in Fig. 11.

### *Ixodes (Pholeoixodes) rugicollis* Schulze and Schlottke

The following location was added to the distribution map of *I. rugicollis*: 1 (Heddergott 2009). A total of seven known locations is depicted in Fig. 9.

### *Ixodes (Pholeoixodes) simplex* Neumann

No location was added to the map of *I. simplex*. The only location known up to now is depicted in Fig. 2.

### *Ixodes (Filippoviella) trianguliceps* Birula

The following five locations were added to the distribution map of the shrew or vole tick *I. trianguliceps*: 2 (Weidner 1954), 1 (Bauch and Danner 1988), 1 (Matuschka 1993), 1 (Walter 2000). A total of 44 out of 54 known locations is depicted in Fig. 7.

### *Ixodes (Ceratixodes) uriae* White

One location was added to the distribution map of the seabird tick *I. uriae*: 1 (Rollins et al. 2025). The current occurrence of *I. uriae* on the North German island of Heligoland confirms the findings from 35 years ago (Liebisch and Vauk-Hentzelt 1992). One out of two known locations is depicted in Fig. 1.

### *Ixodes (Ixodes) ventalloi* Gil Collado

The rabbit tick *Ixodes ventalloi* is the second tick species to be included for the first time in the tick atlas of Germany, and therefore its distribution is briefly described here. The global distribution of *I. ventalloi* extends from the Azores in the Atlantic Ocean (Rosa et al. 2024) to Cyprus in the eastern Mediterranean (Tsatsaris et al. 2016) between 29$^{\circ }$ W–34$^{\circ }$ E and 35–51$^{\circ }$ N. The northernmost occurrences are located in the United Kingdom on Lundy, the Isles of Scilly, and the Channel Islands (Gillingham et al. 2020). However, its main distribution area lies in the western Mediterranean, extending from mainland Portugal through Spain and France to Central Italy. This also includes the North African coast from Morocco through Algeria to Tunisia. In general, the distribution of *I. ventalloi* corresponds well to the original range of its main host, the rabbit *Oryctolagus cuniculus*, which extends across the aforementioned countries. More easterly regions such as the Balkans are free of rabbits and *I. ventalloi* is also not found there. However, rabbits have extended their range, which originally reached as far east as central France, to Germany and beyond. Therefore, a record of *I. ventalloi* described 30 years ago in southwestern Germany near the French border (Beichel et al. 1996; Petney et al. 1996) and a location, at that time published as *Ixodes festai*, were included in the atlas of ticks in Germany. The following two locations are shown in the map of *I. ventalloi*: 1 (Walter et al. 1979), 1 (Petney et al. 1996). Two known locations are mapped in Fig. 3.

### *Ixodes (Pholeoixodes) vespertilionis* Koch

No locations were added to the distribution map of the long-legged bat tick *I. vespertilionis*. A total of 18 out of 19 known locations is depicted in Fig. 2.

### *Ornithodoros (Alectorobius) maritimus* Vermeil and Marquet

The seabird tick *O. maritimus* is the third tick species to be included for the first time in the tick atlas of Germany, and therefore its distribution is briefly described here. The global distribution of soft tick *O. maritimus* extends from the Atlantic coasts of Morocco, Portugal and Ireland to the Aral Sea in Uzbekistan and Kazakhstan between 11$^{\circ }$ W–59$^{\circ }$ E and 31–54$^{\circ }$ N. Most documented findings are located on the coasts of Europe and Northern Africa, often on offshore islands that serve as breeding colonies for seabirds. Documented seabird hosts comprise Alcidae, Fregatidae, Laridae, Pelecanidae, Phalacrocoracidae, Procellariidae, Spheniscidae, Sternidae and Sulidae (Dietrich et al. 2011). The map by Guiguen et al. (1989) shows the most complete global distribution of *O. maritimus*, although more recent findings on Spanish (Sanz-Aguilar 2024), Italian (Fois et al. 2016) and German (Rollins et al. 2025) islands are missing there. The first finding of *O. maritimus* (syn. *Alectorobius maritimus*) in Germany was documented on the island of Heligoland by Rollins et al. (2025) and is also the northernmost documented location worldwide at 54.19$^{\circ }$ N. The only known location is depicted in Fig. 1.

### *Rhipicephalus sanguineus* (Latreille)

The following 32 locations were added to the map of *R. sanguineus* sensu lato: 29 (Fachet-Lehmann et al. 2025), 3 (Köppen et al. 2025). A total of 71 out of 108 known locations is mapped in Fig. 12.

### General discussion

The greatest progress compared to the previous versions of the atlas of ticks in Germany (Rubel et al. 2021, 2023) was made in mapping new find spots of rarely observed tick species such as *C. vespertilionis*, *Ha. concinna*, *Ha. punctata*, *I. lividus*, *I. frontalis*, *I. rugicollis* and *I. trianguliceps*. Furthermore, new species such as the seabird tick *O. maritimus*, the rabbit tick *I. ventalloi* and the introduced tortoise tick *Hy. aegyptium* were included. Before the last three species mentioned were included in the atlas of ticks in Germany, global distribution maps were used to check whether their reported locations in Germany were reliable. The global distribution of *Hy. aegyptium* has already been published, including both natural occurrences and reported findings of imported ticks (Rubel 2025).

The finding of the seabird tick *O. maritimus* was evaluated using an updated distribution map by Guiguen et al. (1989) as described above. According to this, the first finding of *O. maritimus* on Heligoland, collected from common murre *Uria aalge* chicks during the annual ringing effort (Rollins et al. 2025), fits into the previously known distribution pattern of the species. Common murre form huge, dense colonies on the steep cliffs of Heligoland, especially on the famous Lummenfelsen (engl. murre rock), which is the only breeding ground of this species in Germany. According to this, the global distribution area of *I. uriae*, which covers high geographical latitudes around the Arctic and Antarctic, and of *O. maritimus*, which occurs in temperate latitudes such as Europe and Northern Africa, overlaps on the North Sea island of Heligoland. This small island is generally a hot spot for rarely observed tick species. In addition to the two seabird ticks *I. uriae* and *O. maritimus* (Fig. 1), *I. ventalloi* (Fig. 3), *Ha. punctata* (Fig. 5), *Hy. marginatum* (Fig. 6), *I. arboricola* and *I. frontalis* (Fig. 8) as well as *I. ricinus* (Fig. 11) were also reported, which makes a total of eight tick species. With the exception of *I. ricinus* and *I. ventalloi*, all these tick species are associated with seabird breeding colonies or migratory birds.

Evaluating the occurrence of the rabbit tick *I. ventalloi* in Germany proved more difficult, because the tick species was mistakenly described by Arthur (1957) as *Ixodes festai* Tonelli-Rondelli, 1926. Thus, the finding of *I. ventalloi* on Heligoland was published by Walter et al. (1979) under the name *I. festai* according to the determination key of Arthur (1965). However, the report of *I. ventalloi* in Baden-Wuerttemberg (Petney et al. 1996) seems to be reliable, since Bruno Gilot, who provided the morphological clarification regarding *I. ventalloi* and *I. festai* (Gilot and Perez 1978), had been consulted. The specimen reported by Petney et al. (1996) was found on a cat, as is also known from other areas where *Or. cuniculus* and *I. ventalloi* are endemic (Otranto et al. 2017). Thus, the question remains open whether or not *I. ventalloi* is endemic in Germany. The following suggests that the findings of *I. ventalloi* are not isolated cases: (1) All of Germany belongs to the distribution area of the wild rabbit, the main host of *I. ventalloi*. (2) In the state of Baden-Wuerttemberg, where *I. ventalloi* was found, the rabbit population is very dense, as hunting statistics prove. In the 1995/1996 hunting season, more than 13,000 wild rabbits were killed (Baden-Württemberg 2025). Wild rabbits were also released on the island of Heligoland as early as the 18th century (Vauk 1965). (3) The climate at the *I. ventalloi* locations, the Rhine-Main valley, is very mild and known to be home to Mediterranean plants and animals. This includes the thermophilic tick *D. marginatus* (Walter et al. 2016; Rubel et al. 2016), which occurs sympatrically with *I. ventalloi* in western continental Europe and northern Africa. For the reasons mentioned above, it is postulated here that *I. ventalloi* occurs in Germany, although largely undiscovered. This could be due, not least, to the fact that *I. ventalloi* were historically mistakenly identified as *I. festai* or by non-Acaraologists without closer examination as *I. ricinus* (Estrada-Peña et al. 2017). In this context, it should be mentioned that the confusion of *I. ventalloi* and *I. festai* also occurs in current works, as clarified by Hornok et al. (2025), and a global distribution map for *I. festai*, which was mainly found on migratory birds, does not exist, yet.

Another tick species rarely observed in Germany is the sand martin tick *I. lividus*. Although there are no recent studies on this species, previously unconsidered tick locations were described from a historical report of the Zoological Museum Hamburg (Weidner 1954) and from observations made in the former German Democratic Republic (Wadewitz 1955; Schmidt 1990). These are the first and only mapped *I. lividus* observations from the German federal states of Hamburg, Brandenburg and Saxony. For example, almost every examined sand martin nest was infested with ticks on the banks of the Mulde river in Saxony (Wadewitz 1955). Since no more precise location information is available, a colony near Weiditz, where numerous sand martins still breed today (Ritz 2021), was chosen as a representative example of the numerous breeding colonies on the banks of the Mulde river. The first evidence of *I. frontalis* (Lassen et al. 2025) and *I. rugicollis* (Heddergott 2009) was reported in the federal state Thuringia. The map of *Ha. punctata* was also extended to include the first findings both in Hamburg (Weidner 1954) and on the island of Heligoland (Rollins et al. 2025). The map of shrew and vole tick *I. trianguliceps* (Rubel and Kahl 2023) was expanded by five locations and for the relict tick *Ha. concinna* (Rubel et al. 2018) the northernmost findings in Germany, located in the city park of Greifswald, were described by Fischer et al. (2025a).

Since introduced tick species and their potential establishment in Germany due to climate change are currently the focus of research, knowledge on their find spots in Germany has also been significantly expanded. Thus, the map of the *Hyalomma* species (Fig. 6) was extended by many new findings (Chitimia-Dobler et al. 2024b) as well as two historical *Hyalomma* spp. reports (Zeller 1921; Eichler 1934). *Hyalomma marginatum* is originally distributed from the Mediterranean region to western Asia, and *Hy. rufipes* originates from sub-Saharan Africa and regions around the Red Sea (Apanaskevich and Horak 2008). Larvae and nymphs of these two-host ticks have been dispersed throughout Germany and neighboring countries (Romanek et al. 2025; Walder et al. 2026) by migratory birds every spring. For example, *Hy. marginatum* larva and nymph feed on the same host individual and the engorged nymph detaches 3–4 weeks after larval attachment and moults into the adult stage if the local microclimate is warm and dry enough. Rising temperatures in the spring and early summer as a result of climate change have increased the chances for a successful development to the adult stage of *Hy. marginatum*. If the temperatures further increase in the course of the next decades, it may be that *Hy. marginatum* has a chance to become endemic in Central Europe. The winter is no absolute barrier for *Hy. marginatum* as this species occurs in areas in western Asia where the winter temperatures are even colder than in most parts of Central Europe. It should be noted that *Hyalomma* spp. were already observed in Germany a hundred years ago. Paul Schulze identified ticks from migratory birds on the island of Heligoland in 1927 and 1932 as nymphal *Hyalomma* spp. (Eichler 1934) and even before nymphs of the genus *Hyalomma* had already been reported from the island of Norderney (Zeller 1921).

A new study on *R. sanguineus* sensu lato (Fachet-Lehmann et al. 2025), a complex of closely related species (Nava et al. 2015, 2018), confirms the results of earlier studies from the former Federal Republic of Germany, while the results from Köppen et al. (2025) represent the first findings in the East German federal states of Brandenburg and Saxony.

The sporadic detection of further tick specimens, for example on people returning from abroad, is not taken into account here. These include, for example, ticks of the genera *Amblyomma* and *Dermacentor* attached to German travelers returning from the American continent (Chitimia-Dobler et al. 2024a). More often, tick species that specialize in reptiles are introduced through the trade in terrarium animals. In addition to the locations of *Hy. aegyptium* on tortoises shown in Fig. 6, Weidner (1954) lists a total of nine *Amblyomma* species that were found in Hamburg (zoological garden, Hagenbeck’s animal park and private terrariums) on snakes (*Boa constrictor, Python bivittatus*) and lizards (*Iguana iguana, Varanus albigularis, Varanus griseus, Varanus bengalensis*). It is also noteworthy that the introduction of the argasid tick *Ornithodoros talaje* with seabird guano, a popular plant fertilizer from the Pacific islands, was documented in 1898 (Weidner 1954).

The distribution of the two *Dermacentor* species endemic to Germany, *D. marginatus* and *D. reticulatus*, is supplemented by a selection of current findings from citizen science projects (Figs. 3 and 4, depicted in light red). The citizen science data (Springer et al. 2022; Köppen et al. 2025) confirm the known distribution patterns by showing a densification of the locations where the species were found, but also show that *D. reticulatus* is more widespread in Northern Germany than previously assumed (Fischer et al. 2025b). The distribution extends to the border of Denmark, which until recently was considered free of *Dermacentor* populations (Kjær et al. 2019), although the invasion of Denmark by *D. reticulatus* may be imminent, if not already underway. Highly mobile animals such as the golden jackal and the European wolf may accelerate that process (Klitgaard et al. 2017).

Finally, Table 2 provides an insight into the dynamics of tick research in Germany. Although the most common tick species, *I. ricinus*, was already known in the Roman Empire in pre-Christian times (Pagenstecher 1861), Table 2 only begins with the time when the first tick occurrences could be mapped. In the case of *I. ricinus*, that was a finding in a village north of Berlin in 1888 (Nuttall 1916). Even earlier, there was a report about *A. reflexus* in Germany, according to which a large number of ticks were found in the attic of a house in Frankfurt am Main after they had infested the residents (Pagenstecher 1862). The first known locations of *Ha. punctata* (syn. *Rhipicephalus expositicius*) also date back to the 1860 s (Koch 1877) and *Dermacentor* sp. was already described in Germany in 1804 (Hohorst 1943). At that time, all *Dermacentor* species occurring in Germany were classified as *D. marginatus*. It was only a century later that the understanding prevailed that two *Dermacentor* species occur in Germany and that *D. reticulatus* is the more common and also more widely distributed northward. For this reason, the first findings of *D. marginatus* mapped here date from 1925 (Hohorst 1943), and those of *D. reticulatus* from 1959 (Bauch and Danner 1988). Most tick species were first discovered and described in the 1920 s (Schulze 1923, 1924; Schulze and Schlottke 1929) and one species, the seabird argasid tick *O. maritimus*, was found in Germany only in 2024 (Rollins et al. 2025). The exact year as well as references for the first and latest mapped tick locations of all tick species can be found in the supplements (Table S1).

**Table 2:**
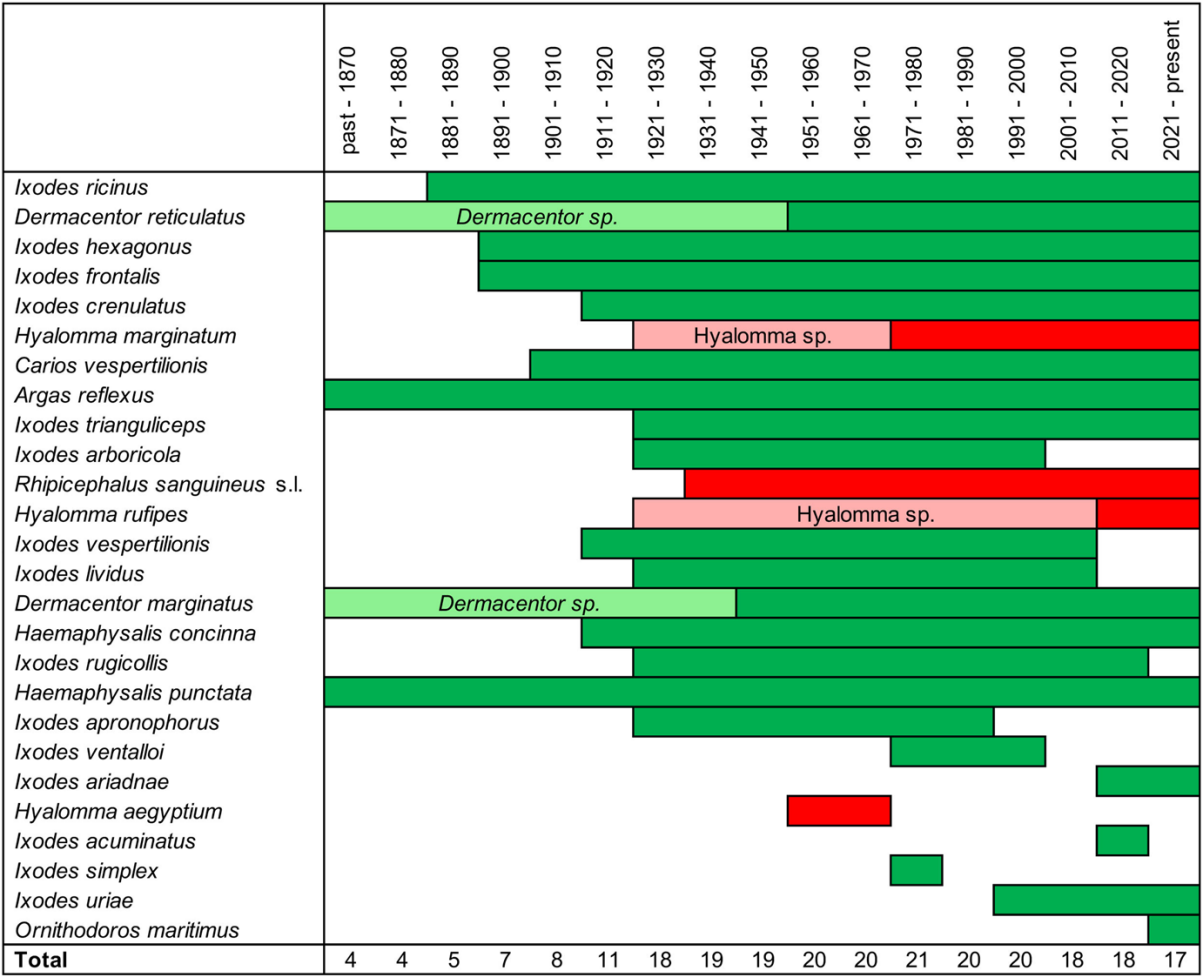
Chronological overview of the 26 tick species in Germany with the first documented locations from the period before 1870, separated into endemic species (green bars) and imported species (red bars). The latter include ticks of the genus *Rhipicephalus* and *Hyalomma*. Early locations where ticks of the genera *Hyalomma* and *Dermacentor* were found, but not identified to the species level, are shown in lighter colours. The order of the tick species corresponds to that in Table 1, with the most widespread species *I. ricinus* on the top of the table and the species *I. simplex*, *I. uriae* and *O. maritimus*, which were only observed at one location, at the bottom of the table

## Conclusions

The second data update of the atlas of ticks in Germany is presented here. Greatest progress compared to the previous versions was made in mapping the find spots of rarely observed species (*C. vespertilionis*, *Ha. concinna*, *Ha. punctata*, *I. arboricola*, *I. frontalis*, *I. lividus*, *I. rugicollis*, *I. trianguliceps*), species that were mapped for the first time in Germany (*I. ventalloi*, *O. maritimus*) and continuously introduced species (*Hyalomma* spp., *R. sanguineus* s.l.). However, new locations of common tick species such as *D. marginatus*, *D. reticulatus*, *I. crenulatus*, *I. hexagonus* and *I. ricinus* were also added. This leads to a better knowledge of the tick fauna in the individual federal states. For example, the number of mapped tick species in Saxony has increased by six. Thus, the second data update with the underlying digital dataset in the supplement offer the most comprehensive map material to date on the distribution of tick species in Germany. Of the 26 tick species ever detected in Germany, 21 were observed in the period 2001–present. Endemic tick species not currently detected are mostly endophilic ixodid ticks, which cannot be found by flagging. One needs to check their hosts for feeding ticks or investigate the dens/nests or wintering grounds for off-host ticks. Therefore, missing findings of endophilic ticks in recent decades do not necessarily indicate the current absence of those tick species, but merely show that no field studies on those ticks have been conducted in the recent past. For example, to carry out studies on bats (infested by *I. vespertilionis, I. simplex, I. ariadnae*) and sand martins (infested by *I. lividus*) is difficult in Germany because they are strictly protected animals.

## Supplementary Information

Below is the link to the electronic supplementary material.

Supplementary file 1 (pdf 45 KB)

Supplementary file 2 (xlsx 55 KB)

## Data Availability

All data supporting the findings of this study are available within the paper and its Supplementary Information.
