# Supplementary material for: Atlas of ticks (Acari: Argasidae, Ixodidae) in Germany: 2nd data update with focus on introduced and rarely observed species
Source: Exp Appl Acarol. 2026 Jul 23;97(2):18. doi: 10.1007/s10493-026-01168-1 (PMC13396030; doi:10.1007/s10493-026-01168-1)
Supplement: Supplementary file 1 — (pdf 45 KB) [file 10493_2026_1168_MOESM1_ESM.pdf]

Supplementary Material

Atlas of ticks (Acari: Argasidae, Ixodidae) in Germany: 2nd data update with focus on introduced and rarely observed species

Table S1 First and latest findings of the 26 tick species mapped in Germany.

No.	Species	First Finds	Latest Finds	References
1	*Ixodes ricinus*	1888	2025	Nuttall (1916), Krüger et al. (2025)
2	*Dermacentor reticulatus*	1959	2025	Bauch and Danner (1988), Fischer et al. (2025)
3	*Ixodes hexagonus*	1899	2025	Weidner (1954), Krüger et al. (2025)
4	*Ixodes frontalis*	1894	2025	Weidner (1954), Krüger et al. (2025)
5	*Ixodes canisuga*	1894	2023	Schulze (1918), Schantz et al. (2025)
6	*Hyalomma marginatum*	1894	2024	Walter et al. (1979), Chitimia-Dobler et al. (2024)
7	*Carios vespertilionis*	1901	2022	Voigts and Oudemans (1904), Fritzsche et al. (2023)
8	*Argas reflexus*	1862	2025	Pagenstecher (1862), Köppen et al. (2025)
9	*Ixodes trianguliceps*	1929	2023	Weidner (1954), Weigand et al. (2023)
10	*Ixodes arboricola*	1929	1991	Schulze and Schlottke (1929), Walter (1992)
11	*Rhipicephalus sanguineus*	1937	2024	Eichler et al. (1968), Fachet-Lehmann et al. (2025)
12	*Hyalomma rufipes*	2015	2024	Chitimia-Dobler et al. (2016, 2024)
13	*Ixodes vespertilionis*	1914	2004	Lengersdorf (1929), Rupp et al. (2004)
14	*Ixodes lividus*	1929	2010	Schulze and Schlottke (1929), Klaus et al. (2016)
15	*Dermacentor marginatus*	1925	2025	Hohorst (1943), Krüger et al. (2025)
16	*Haemaphysalis concinna*	1912	2025	Schulze (1923), Fischer et al. (2025)
17	*Ixodes rugicollis*	1929	2015	Schulze and Schlottke (1929), Kretschmar (2016)
18	*Haemaphysalis punctata*	1869	2024	Koch (1877), Rollins et al. (2025)
19	*Ixodes apronophorus*	1924	1989	Schulze (1924), Olbrich and Liebisch (1991)
20	*Ixodes ventalloi*	1979	1994	Walter et al. (1979), Petney et al. (1996)
21	*Ixodes ariadnae*	2015	2023	Hornok et al. (2015), Weigand et al. (2023)
22	*Hyalomma aegyptium*	1954	1961	Weidner (1954), Negrobov and Borodin (1964)
23	*Ixodes acuminatus*	2012	2012	Petney et al. (2015)
24	*Ixodes simplex*	1972	1972	Walter and Kock (1985)
25	*Ixodes uriae*	1992	2024	Liebisch and Vauk-Hentzelt (1992), Rollins et al. (2025)
26	*Ornithodoros maritimus*	2024	2024	Rollins et al. (2025)

References

Bauch, R. J., Danner, G., 1988. The discovery of *Dermacentor reticulatus* (Ixodida, Ixodidae) in the East German districts of Leipzig and Halle (in German). Angew. Parasitol. 29, 250–254.

Chitimia-Dobler, L., Nava, S., Bestehorn, M., Dobler, G., Wölfel, S., 2016. First detection of *Hyalomma rufipes* in Germany. Ticks Tick Borne Dis. 7, 1135–1138.

Chitimia-Dobler, L., Springer, A., Lang, D., Lindau, A., Fachet, K., Dobler, G., Nijhof, A. M., Strube, C., Mackenstedt, U., 2024. Molting incidents of *Hyalomma* spp. carrying human pathogens in Germany under different weather conditions. Parasit. Vectors 17, 70.

Eichler, W. D., Arthur, D. R., Hoogstraal, H., Lachmajer, J., 1968. Kritische Liste mitteleuropäischer Zeckenarten (in German). Angew. Parasitol. 9, 88–97.

Fachet-Lehmann, K., Lindau, A., Mackenstedt, U., 2025. Unwanted souvenirs–import routes and pathogen detection of the non-endemic tick *Rhipicephalus sanguineus* s.l. in Germany. Exp. Appl. Acarol. 94, 42.

Fischer, S., Drewes, S., Sägert, E., Silaghi, C., 2025. Ticks and tick-borne pathogens in the city park of Greifswald, an urban area of Mecklenburg-Western Pomerania, in 2022. Proc. 16th Int. Symp. Ticks Tick-borne Dis., 26 to 28 March 2025, Weimar, Germany.

Fritzsche, A., Zaenker, S., Gottwald, J., Keil, R., Zaenker, C., Bröker, M., Chitimia-Dobler, L., 2023. Distribution of the soft tick *Carios vespertilionis* in lowlands and low mountain regions of Germany. Exp. Appl. Acarol. 91, 89–97, https://doi.org/10.1007/s10493-023-00822-2.

Hohorst, W., 1943. Die Zecke *Dermacentor marginatus* Sulzer 1776, ihre Verbreitung, Lebensweise und medizinische Bedeutung (in German). Z. Parasitenk. 13, 118–146.

Hornok, S., Takács, N., Szoke, K., Kunz, B., 2015. First record of *Ixodes ariadnae* in Germany. Acta Vet. Hungarica 63, 347–351.

Klaus, C., Gethmann, J., Hoffmann, B., Ziegler, U., Heller, M., Beer, M., 2016. Tick infestation in birds and prevalence of pathogens in ticks collected from different places in Germany. Parasitol. Res. 115, 2729–2740.

Koch, L., 1877. Verzeichnis der bei Nürnberg bis jetzt beobachteten Arachniden (mit Ausschluss der Ixodiden und Acariden) und Beschreibung von neuen hier vorkommenden Arten (in German). Abhandl. Naturhist. Ges. Nürnberg 6, 113–198.

Köppen, K., Zmarlak-Feher, N. M., Dörre, A., Hagedorn, P., Kohl, C., Heuner, K., 2025. Country-wide assessment of tick-borne pathogens collected in ticks between 2021 and 2024 in Germany, with a focus on *Francisella*: A one health pilot study. One Health 21, 101190.

Kretschmar, F. M., 2016. Die Parasiten des Europäischen Iltisses *Mustela putorius* Linnaeus, 1758 in Deutschland (in German). Doctoral thesis, Univ. Munich, 194pp.

Krüger, A., Berweiler, S., Wolff, J., Klinger, A., Schummel, T., Hagen, R. M., Scheid, P. L., 2025. Flagging records of *Ixodes frontalis* (Panzer, 1798) and *Dermacentor marginatus* (Sulzer, 1776) (Acari: Ixodidae), and their first reporting from Coblenz region, Western Germany. Exp. Appl. Acarol. 94, 13.

Lengersdorf, F., 1929. Beitrag zur Kenntnis der Höhlenfauna Westfalens (in German). Verhandlungen des naturhistorischen Vereines der preussischen Rheinlande 85, 106–108.

Liebisch, A., Vauk-Hentzelt, E., 1992. The first record of the tick species *Ixodes (Ceratoxides) uriae* White, 1852 in Germany. Int. J. Med. Microbiol. Hyg. 325, 52–53.

Negrobov, V. P., Borodin, V. S., 1964. Einige seltene Zeckenfunde im mittleren Teil der DDR (in German). Angew. Parasitol. 5, 107–111.

Nuttall, G. H. F., 1916. Notes on ticks. IV. Relating to the genus *Ixodes* and including a description of three new species and two new varieties. Parasitology 8, 294–337.

Olbrich, S., Liebisch, A., 1991. Epidemiological studies of the infection of ticks with borreliosis agents in small mammals from North Germany (in German). Dtsch. tierärztl. Wschr. 98, 228–230.

Pagenstecher, H. A., 1862. Zur Anatomie von *Argas reflexus* (in German). Z. wissensch. Zool. 11, 142–155, https://www.zobodat.at (accessed on 10 Feb. 2026).

Petney, T. N., Beichel, E., Maiwald, M., Hassler, D., 1996. *Ixodes ventalloi*: a new tick record for Germany. Appl. Parasitol. 37, 96–98.

Petney, T. N., Moser, E., Littwin, N., Pfäffle, M., Muders, S. V., Taraschewski, H., 2015. Additions to the 'Annotated Checklist of the Ticks of Germany': *Ixodes acuminatus* and *Ixodes inopinatus*. System. Appl. Acarol. 20, 221–224.

Rollins, R. E., Dierschke, J., Obiegala, A., von Buttlar, H., Chitimia-Dobler, L., Liedvogel, M., 2025. Analysis of ticks (Acari: Ixodida) and associated microorganisms collected on the North Sea Island of Heligoland. Parasitol. Res. 124, 34.

Rupp, D., Zahn, A., Ludwig, P., 2004. Actual records of bat ectoparasites in Bavaria (Germany). Spixiana 27, 185–190.

Schantz, A. V., Stutz, R., Steinhoff, A., Peter, N., Klimpel, S., 2025. Metazoan parasite fauna of the American mink (*Neogale vison*) in comparison with the closely related European mink (*Mustela lutreola*) in Europe. Parasitol. Res. 124, 92.

Schulze, P., 1918. Die Neuheiten der märkischen Insektenfauna 1917 (einschließlich Eriophyiden und Ixodiden) (in German). Deutsch. Ent. Zeitschr. 1918/1919, 277–292.

Schulze, P., 1923. *Haemaphysalis concinna* Koch (Ixod.) in Brandenburg (in German). Deutsch. Ent. Zeitschr. 1923, 612–612.

Schulze, P., 1924. *Ixodes apronophorus* n. sp., eine neue deutsche Zecke von *Arvicola amphibius* L. (in German). Zool. Anzeig. 59, 281–284.

Schulze, P., Schlottke, E., 1929. Kleinhöhlenbewohnende deutsche Zecken mit Beschreibung dreier neuer Baumhöhlenbrüter und einer Bestimmungstabelle der deutschen *Ixodes* (in German). Sitzungsber. Abh. Naturf. Ges. Rostock III. F. 2, 95-112.

Voigts, H., Oudemans, A. C., 1904. Zur Kenntnis der Milben-Fauna von Bremen (in German). Abhandl. Naturw. Verein Bremen 18, 199–253.
URL `www.zobodat.at`

Walter, G., 1992. Verbreitung und Biologie von *Argas vespertilionis*, *Ixodes simplex* und *Ixodes ricinus* (Ixodidea: Ixodidae; Argasidae) bei Fledermäusen (Chiroptera) in der Bundesrepublik Deutschland (in German). Myotis 30, 123–132.

Walter, G., Kock, D., 1985. Records of *Ixodes vespertilionis*, *I. simplex* and *Argas vespertilionis* (Ixodoidea: Ixodidae, Argasidae) from German bats (Chiroptera). Z. Parasitenk. 71, 107–111.

Walter, G., Liebisch, A., Vauk, G., 1979. Untersuchungen zur Biologie und Verbreitung von Zecken (Ixodoidea, Ixodidae) in Norddeutschland. II. Zecken der Zugvögel auf der Insel Helgoland (in German). Z. Angew. Zool. 667, 445–4618.

Weidner, H., 1954. Die Pseudoskorpione, Weberknechte und Milben der Umgebung von Hamburg (in German). Entomol. Mitt. Zool Staatsinst. Zool. Museum Hamburg, 4, 105–165.

Weigand, A., Zaenker, S., Weber, D., Schaper, S., Bröker, M., Zaenker, C., Chitimia-Dobler, L., 2023. Tick findings from subterranean environments in the Central German Uplands and Luxembourg reveal a predominance of male *Ixodes hexagonus*. Exp. Appl. Acarol. 89, 461–473.
